# Editorial: Specialized metabolites and stress resistance of forest trees

**DOI:** 10.3389/fpls.2023.1211750

**Published:** 2023-05-31

**Authors:** Niu Yu, Shengqing Shi, Tao Yao, Hai-Xi Sun

**Affiliations:** ^1^ State Key Laboratory of Tree Genetics and Breeding, Research Institute of Tropical Forestry, Chinese Academy of Forestry, Guangzhou, China; ^2^ State Key Laboratory of Tree Genetics and Breeding, Key Laboratory of Tree Breeding and Cultivation of National Forestry and Grassland Administration, Chinese Academy of Forestry, Beijing, China; ^3^ Biosciences Division, Oak Ridge National Laboratory, Oak Ridge, TN, United States; ^4^ BGI-Shenzhen, Shenzhen, China

**Keywords:** specialized metabolites, abiotic stresses, terpenes, transcriptional regulation, transcription factor

Forest trees consistently confront various biotic and abiotic stresses throughout their lifespan and they have evolved specialized metabolites in addition to primary metabolites to resist these threats. The internal physiological and molecular mechanisms are crucial for understanding the function of metabolites in response to severe stresses. Terpenes constitute the largest class of plant secondary metabolites, which play a role in various biological and ecological processes and are also used in the production of valuable tree-based oils. Although the terpene biosynthesis pathway is well characterized, its transcriptional regulation is not well understood. Yang et al. systematically investigated the distribution and expression patterns of the bHLH transcription factor family in *Lisea cubeba* and identified candidate genes that contribute to monoterpene biosynthesis as potential regulators. This led to the discovery and validation of *LcbHLH78* in promoting geraniol and linalol biosynthesis. Ren et al. utilized the expression patterns and co-expression network analysis to show that *Ts WRKYs* potentially play a regulatory role in terpene biosynthesis. While the regulatory pathway of terpene biosynthesis is worthy of further investigation, the biological roles of terpene, especially in the resistance to stresses, still need more convincing and common evidence from distinctive trees.

Specialized metabolites are derived from primary metabolites and their functions are closely related to the essential nutrient element. Potassium ions are primarily found in the cytoplasm and are involved in various metabolic processes. However, little is known about how potassium deficiency regulates the transcriptome and metabolome of plants, particularly in coconut palms. In a paper published by Lu et al. on this Research Topic, they found that potassium deficiency leads to reduced height, biomass, soluble protein, and soluble sugar content in coconut seedlings. By integrating transcriptomic and metabolomic analyses, the authors identified numerous pathways that respond to potassium deficiency stress including terpene biosynthetic process. These findings are consistent with previously reported in other annual plants (Ding et al., 2021, Plant Cell Environ. 44, 186–202). Nevertheless, new and unique response processes are expected in perennial trees.

Condensed tannins (CTs) (also referred to as proanthocyanidins (PAs)) are plant-defense-related secondary metabolites of phenolic compound commonly found in woody plants and forest trees. It has been shown that the biosynthesis of CTs protects against herbivores and rust infections in *Populus*. However, their ecosystem functions and genotypic effects have not been evaluated in forest environments. Siddique et al. studied the autochthonous relationship between *P. tremula* and *Melampsora pinitorqua* by assessing CT contents and fungal markers, revealing the functions of CTs and the genotypic effects in forest environments. On the other hand, although many chromatographic methods could allow the detection of PAs accurately, they are unsuitable for determining *in situ* PA levels at the cellular level. Chowdhury et al. utilized the fluorogenic properties of 4-dimethylaminocinnamaldehyde (DMACA) to develop a useful approach for determining the localization and dynamics of PAs in plant tissues and cellular compartments. This method will greatly help the localization of both intracellular and cell-wall-bound PAs in plants.

Considering the diversity, abundance, and specificity, the biological function of specialized metabolites in response to the environment may be related to the natural properties and need deeper research from various plants that accumulate special metabolites. With the advance of big data era, we are expecting new and interesting insights with respect to the specialized metabolites in forest trees.

## Author contributions

NY initiated the Research Topic. SS, TY and H-XS edited the submitted papers. All authors contributed to the article and approved the submitted version.

